# Ascertaining the mechanistic etiology of COVID-associated glomerulonephritis: a systematic review

**DOI:** 10.3389/fmed.2025.1568943

**Published:** 2025-06-09

**Authors:** Brendan M. Coyne, Danielle Ito, Anam Tariq, Susie Q. Lew, Jeffrey Kopp, Patricia Centron Vinales, Fahim Malik, Patrick E. Gipson, Ehsan Nobakht

**Affiliations:** ^1^George Washington School of Medicine and Health Sciences, Washington, DC, United States; ^2^Department of Biological Sciences, University of California, Irvine, Irvine, CA, United States; ^3^National Institute of Diabetes and Digestive and Kidney Diseases, National Institute of Health, Bethesda, MD, United States; ^4^Washington Nephrology Associates, Rockville, MD, United States

**Keywords:** COVID-19, glomerulonephritis, glomerulopathies, COVID-19 associated nephropathy, immunology, immune complex, cytokines, vaccines

## Abstract

**Background:**

Since its first reported case in December 2019, COVID-19 disease, caused by severe acute respiratory coronavirus 2 (SARS-CoV-2), evolved into a major pandemic throughout the world. Although COVID-19 is most often characterized as a respiratory pathology, there are also extensive reports of renal complications, such as glomerulonephritis (GN). However, the precise nature of COVID-associated glomerulonephritis (COVID-GN) has yet to be fully understood. This review seeks to elucidate COVID-GN pathophysiology by conducting an exhaustive systematic review.

**Methods:**

Herein, we compare the different GN subtypes associated with COVID-19 in the literature. We also review the cytokines, antibodies, and genes most implicated in COVID-GN.

**Results:**

The GN subtype with the highest number of cases associated with COVID-19 infection was focal segmental glomerulosclerosis, specifically the collapsing morphology. Meanwhile, the highest number of cases associated with COVID-19 vaccination was IgA nephropathy. The most prevalent mechanism in the literature for COVID-GN involves a cytokine storm, which may be accompanied by immune complex deposition.

**Discussion:**

Both infection and vaccination from SARS-CoV-2 can induce robust CD4+ T cell responses promoted by an IL-6 amplifier loop of inflammation. This immune response is likely further enhanced by interactions with complement systems and the renin-angiotensin-aldosterone system (RAAS). SARS-CoV-2-mediated pathways of both direct cytotoxicity and stimulation of polyclonal immunoglobulin may converge to cause glomerular inflammation and injury. Further investigation of these inflammatory pathways may provide insight into COVID-19 pathophysiology, treatment, and long-term outcomes.

## 1 Introduction

Since its first reported cases of human infection in December 2019, the severe acute respiratory coronavirus 2 (SARS-CoV-2) pathogen has spread throughout every major country in the world (1). This caused a global health crisis and pandemic, with at least 81 million reported cases and over 1,777,000 deaths within the first year alone (2).

Severe acute respiratory coronavirus 2, similar to other viruses of the Coronaviridae family, enters host cells via binding to angiotensin-converting enzyme (ACE)-2 receptors and subsequent endocytosis and/or membrane fusion of the receptor-virus complex (3, 4). Binding to ACE-2 is dependent on SARS-CoV-2’s surface spike protein (S), which is proteolytically activated by host transmembrane protease serine 2 (TMPRSS2) (5). SARS-CoV-2’s ribonucleic acid (RNA) genome is replicated and translated within the cytoplasm forming new SARS-COV-2 virions (6). Furin-mediated processing of S on the virion’s surface is followed by release of the newly-formed viruses into the extracellular environment (6). This viral life cycle perpetuates throughout the body, targeting vital organs (7).

Severe acute respiratory coronavirus 2 infection can cause COVID-19, which is most often characterized as a respiratory disease (8). However, renal involvement and substantial acute kidney injury (AKI) may also occur (9). A high proportion of hospitalized COVID-19 patients present with AKI, proteinuria, and/or hematuria, suggesting glomerular or tubulointerstitial involvement (10).

Coronavirus disease 2019’s connection to renal tubular damage is well-established and comprises the majority of COVID-related AKI (9). However, glomerulonephritis (GN) is also an important complication, and its association with SARS-CoV-2 remains to be fully understood. GN appears to be one of the most destructive renal pathologies in COVID-19; COVID-19 vaccine-associated GN had higher fatality than either vaccine-associated AKI or tubulointerstitial nephritis (TIN) (11). However, there remains much uncertainty regarding the precise nature of this relationship. There is some doubt as to whether the perceived association between SARS-CoV-2 and GN is causal versus coincidental. Thus, ongoing surveillance of COVID-associated GN (COVID-GN) would be prudent as SARS-CoV-2 and its variants continue to circulate throughout the world.

## 2 Methods

A comprehensive review with a timeline from 1 January 2020 to 31 December 2023 was conducted in accordance with Preferred Reported Items for Systematic Reviews and Meta-Analysis (PRISMA) guidelines (12).

### 2.1 Study selection

A digital research database search was performed with the following key terms: COVID-19, SARS-CoV-2, glomerulonephritis, nephropathy, and nephritis. Literature was searched within four electronic databases (PubMed, MEDLINE, EBSCO, Scopus, and Google Scholar) during the time frame of 1 January 2020 through 31 December 2023.

Authors BC and DI independently screened studies by title, abstract, and full text. Relevant literature included case reports and series, observational/cohort studies, and meta-analyses. Systematic reviews, editorials, conference abstracts, experimental studies on animal models, and articles without full-text or original data available were excluded. Additional exclusion criteria included: lack of evidence of glomerulonephritis on labs or biopsy, studies describing an unspecified glomerulonephritis, and non-English articles without official translations.

### 2.2 Data extraction

Investigators BC and DI independently compiled key data from the included articles in a standardized Excel spreadsheet. Extracted data was then validated by all authors. Information was collected regarding author lists, publication year, country or region where the study was conducted, disease course, clinical features (e.g., serology, kidney pathology), and outcomes (i.e., morbidity, mortality, complications).

**FIGURE 1:**
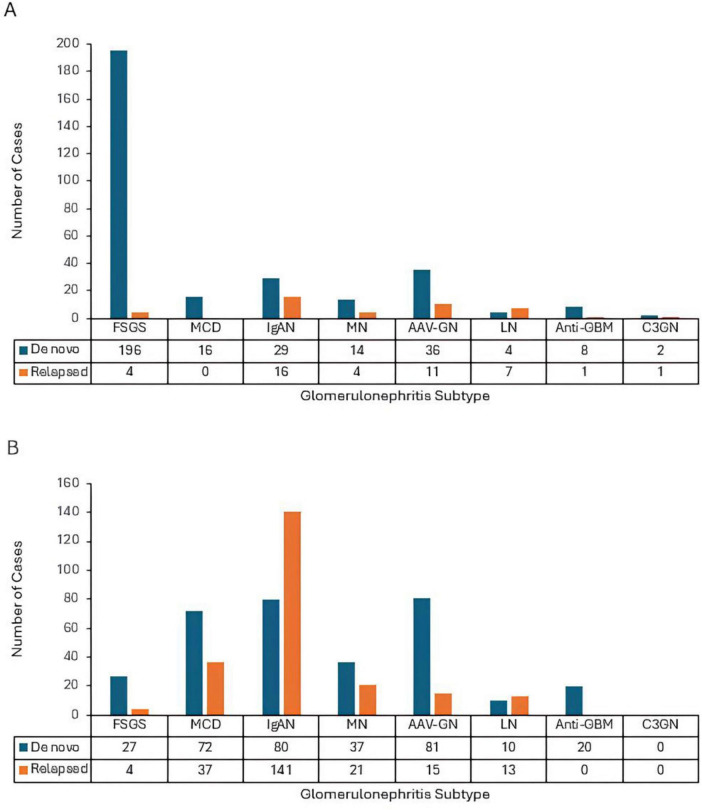
**(A)** Tallied cases of glomerulonephritis (GNs) associated with coronavirus disease 2019 (COVID-19) infection. The highest number of reports was *de novo* FSGS with 196 cases. The lowest frequency was relapsed MCD with 0 reported cases. **(B)** Tallied cases of GNs associated with COVID-19 vaccination. The highest number of reports was relapsed IgAN with 141 cases. The lowest frequency was acute and relapsed C3GN and relapsed anti- GBM with 0 reported cases. FSGS, focal segmental glomerulosclerosis; MCD, minimal change disease; IgAN, IgA nephropathy; MN, membranous nephropathy; AAV-GN, ANCA, associated vasculitis and glomerulonephritis; LN, lupus nephritis; anti-GBM, anti-glomerular basement membrane nephritis; C3GN, C3 glomerulonephritis.

The number was tallied for cases of IgA nephropathy, anti-glomerular basement membrane disease, membranous nephropathy, C3 glomerulonephritis, ANCA-associated vasculitis and glomerulonephritis, lupus nephritis, focal segmental glomerulosclerosis, and minimal change diseases that had a temporal association with SARS-CoV-2 infection or immunization (Figure 1). Each case was further categorized by the presentation, as either acute, *de novo* GN versus a flared relapse of underlying GN.

## 3 Results

### 3.1 Antibody and immune complex formation

**FIGURE 2:**
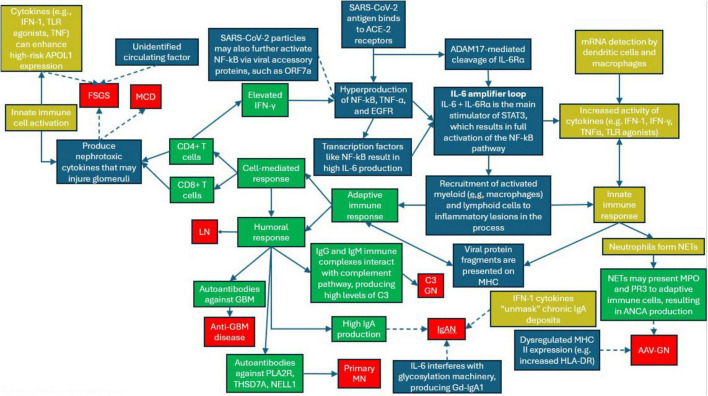
Pathophysiologic summary of coronavirus disease (COVID)-associated glomerulonephritis (GNs). Green boxes represent primarily adaptive immune processes, yellow boxes represent primarily innate immune responses, blue boxes are neutral, red boxes indicate GN subtypes. Mechanisms that are more well-established in the literature are represented by solid arrows, whereas mechanisms that are less well-understood are represented by dashed arrows. IFN-y, interferon gamma; NF-kB, nuclear factor kappa B; TNF-a, tumor necrosis factor a; EGFR, epidermal-like growth factor receptor; STAT3, signal transducer and activator of transcription 3; ORF7a, open reading frame 7a; IL-6, interleukin 6; IL-6Ra, interleukin 6 receptor alpha; TLR, toll-like receptor; ACE-2, angiotensin-converting enzyme 2; ADAM 17, disintegrin and metalloproteinase 17; MHC, major histocompatibility complex; HLA-DR, human leukocyte antigen-DR; THSD7A, thrombospondin type-1 domain- containing 7A; NELL1, nerve epidermal growth factor- like antigen 1; Gd-lgA1, galactose-deficient lgA1; MPO, myeloperoxidase; PR3, proteinase 3.

It is well-established that SARS-CoV-2 and its vaccines can elicit robust cell-mediated and antibody-mediated immune responses (13). This can induce immune dysregulation, manifesting as non-specific immune activation and/or incitation or exacerbation of autoimmune state (14–17). This immune dysregulation appears to be a core mechanism in COVID-GN (Figure 2). The precise means by which this develops remains unclear, although some potential avenues have been explored, such as SARS-CoV-2-mediated cross-reactivity in antibodies and T cells (18–21).

#### 3.1.1 IgA nephropathy

One of the common types of GN investigated is IgA nephropathy (IgAN). COVID-19 disease has shown to potentially evoke and/or exacerbate IgA vasculitis and IgAN (22–44). COVID-19 vaccines have also been associated with both acute IgAN (16, 36, 45–68) and flares of pre-existing IgAN (53, 57, 69–89).

IgA nephropathy represents an autoimmune state that classically follows mucosal infections, in which IgA is upregulated by the immune system and can subsequently deposit into tissues such as the glomerular mesangium (90). Given that SARS-CoV-2 antigens act on receptors widely expressed throughout respiratory and GI mucosa (3), it is quite conceivable how IgAN may follow COVID-19 infection or immunization.

It is possible that COVID-19 exposure activates B cells to produce especially high levels of IgA, which form acute immune complexes that deposit in glomeruli (22, 91). Indeed, the first immunoglobulin detected in COVID-19, anti-SARS-CoV-2 IgA, precedes both IgM and IgG serology (92). This may implicate IgA as one of the predominant agents mediating COVID-19 glomerular damage. However, it is debated as to whether this is attributed to acute IgA production or pre-existing IgA deposits in the glomerular mesangium that are simply “unmasked” by SARS-CoV-2 (70, 74).

Of note, some COVID-associated IgAN cases present with elevated galactose-deficient-IgA1 (Gd-IgA1) in the serum or Gd-IgA1 mesangial deposits (65, 93). Mesangial deposition of Gd-IgA1, an IgA variant caused by aberrant glycosylation, is believed to play a role in IgAN (94). Recognition of SARS-CoV-2 antigens by Gd-IgA1 may result in immune complex and complement deposition in the glomerular mesangium (93). Future research on the induction of Gd-IgA1 and anti-glycan immune complexes may provide insight into these processes.

#### 3.1.2 Anti-glomerular basement membrane disease

Another autoimmune nephropathy associated with COVID-19 is anti-GBM disease (also known as Goodpasture syndrome). Certain cases of COVID-19 disease or COVID-19 vaccines may be associated with anti-GBM nephritis, usually presenting with crescentic, rapidly progressive glomerulonephritis (16, 41, 44, 52, 55, 74, 95–105). This is correlated by a significantly increased incidence of anti-GBM disease since the start of the COVID-19 pandemic (106).

Goodpasture syndrome is classically described as an autoimmune disorder driven by molecular mimicry and autoantibodies (107). Similarly to Streptococcus- and influenza-induced anti-GBM nephritis, the severe endothelial damage from SARS-CoV-2 could conceivably expose basement membrane antigens (e.g., Goodpasture antigen) in the respiratory tract and renal tissue; epitope similarity would then cause autoantibodies to attack glomeruli (96, 108). However, given that the literature remains sparse, the causal relationship between COVID-19 and anti-GBM disease remains speculative.

#### 3.1.3 Membranous Nephropathy

Severe acute respiratory coronavirus 2 infection has been implicated in potentially inducing or exacerbating primary and secondary membranous nephropathy (MN) (35, 44, 96, 109–111). Additionally, immunization with COVID-19 vaccines was associated with both acute (36, 47, 48, 52, 53, 55, 112–117) and relapsed MN (16, 78, 82, 83, 118, 119).

Primary MN often results from autoimmune activity, usually caused by immune complex deposition and presenting with autoantibodies against phospholipase A2 receptor (PLA2R), although autoantibodies against thrombospondin type-1 domain-containing 7A (THSD7A) and nerve epidermal growth factor-like antigen 1 (NELL1) have been implicated as well (120, 121). These antibodies form immune complexes which deposit in sub-epithelial tissue of glomeruli, causing basement membrane thickening (122). Although classically associated with primary MN, PLA2R serologies have also been detected in secondary MN with viral etiology (e.g., hepatitis B, hepatitis C) (120).

It is possible that both primary and secondary MN associated with SARS-CoV-2 involve virus-evoked anti-PLA2R complexes (123). Although some cases test positive for THSD7A or NELL-1 (16, 113), the majority are PLA2R+ (16, 109, 118). Seeing as how PLA2R is expressed in the respiratory tract, SARS-CoV-2-mediated damage may release PLA2R from respiratory epithelial cells, triggering the development of autoantibodies that attack glomerular tissue (123).

However, COVID-19 vaccine-associated MN cases have also tested positive for anti-PLA2R in the serum and/or renal tissue (16, 36, 47, 48, 52, 78, 112, 114, 118). This suggests that direct respiratory damage from SARS-CoV-2 virus is not necessarily required to induce PLA2R+ MN. Rather, messenger RNA (mRNA) or surface antigen may somehow diminish immune tolerance for PLA2R antigen (118), but this hypothesis requires further evidence.

#### 3.1.4 C3 Glomerulonephritis

There are some cases reporting a potential association between SARS-CoV-2 infection and C3 glomerulonephritis (C3GN) (124–126). C3GN is a pathology formerly categorized as a subtype of membranoproliferative glomerulonephritis (MPGN), which is now used primarily to describe glomerular injury patterns as opposed to a specific disease diagnosis (127, 128). A process closely related to C3GN, immune complex MPGN (IC-MPGN) has also been reported to follow SARS-CoV-2 infection or vaccination (35, 47, 78, 124, 125, 129, 130). One case exhibited lupus-like features with a “full house” pattern on immunofluorescence (129). Such findings are comparable to the lupus-like biopsies in human immunodeficiency virus (HIV)-associated immune complex kidney disease (HIVICK), which is similarly common among White patients (131).

In HIVICK, it is proposed that systemic and intrarenal immune activity triggers polyclonal B cells and hypergammaglobulinemia with resultant glomerular hyperplasia (132, 133). This mechanism may similarly underlie the strong IgG and IgM staining that was observed in a patient with COVID-19 and C3GN (126). This was correlated with the eventual development of tubuloreticular inclusions (“interferon footprints”), possibly indicating a strong contributory role of interferon (IFN) cytokines (126).

However, research in this area is limited, and there does not seem to be substantial evidence that COVID-19 can trigger C3GN. It is important to recognize that neither C3GN nor IC-MPGN demonstrate consistent, strong associations with COVID-19. However, identification of their pathophysiology may guide researchers toward which mechanisms are more likely or less likely underpinning COVID-GN.

#### 3.1.5 Vasculitis-associated glomerulonephritis

Coronavirus disease 2019 has been previously associated with autoimmune destruction of glomerular blood vessels, which can cause crescentic, necrotizing GN with fibrinoid necrosis (134, 135). Biopsies typically stain negative or very low for immune complexes; hence the disease is often referred to as Pauci-immune GN (136).

Anti-neutrophil cytoplasmic antibody (ANCA)-associated vasculitis glomerulonephritis (AAV-GN) is a group of disorders sharing similar features on biopsy (e.g., often crescentic, Pauci-immune) and classically driven by autoantibodies against neutrophil proteins. Variations of AAV-GN include granulomatosis with polyangiitis (GPA), microscopic polyangiitis (MPA), and eosinophilic granulomatosis with polyangiitis (formerly Churg-Strauss syndrome).

Coronavirus disease 2019 disease has been associated with new-onset GPA (137–139), relapsed GPA (140), and new-onset MPA (141). COVID-19 vaccination has also been temporally linked to new-onset GPA (142, 143), and both new-onset and relapsed MPA (82, 144, 145). Other variations of AAV-GN have also developed following SARS-CoV-2 infection and immunization (16, 17, 36, 38, 44, 47, 48, 55, 78, 99, 100, 146–180).

The hypothesized mechanism for COVID-associated AAV-GN involves COVID-triggered immune dysregulation; specifically, it is possible that SARS-CoV-2 proteins promote the development of autoantibodies against neutrophil proteins such as myeloperoxidase (MPO) and proteinase 3 (PR3), which is characteristic of classic AAV-GN (178, 180–182). The pathophysiology of AAV strongly implicates the role of neutrophil extracellular traps (NETs) as sources of autoantigens, presenting MPO and PR3 to adaptive immune cells (183). NETs normally play a significant role in host defense, but NET dysregulation can lead to angiopathy and ANCA development (184). NETs have been implicated as a critical player in SARS-CoV-2 infection and the cytokine storm that can follow (185, 186); they are also present in kidney biopsies of some COVID-19 patients (187). Autoantigen presentation by NETs is thus a possible mechanism for COVID-associated AAV-GN (178, 188).

**FIGURE 3:**
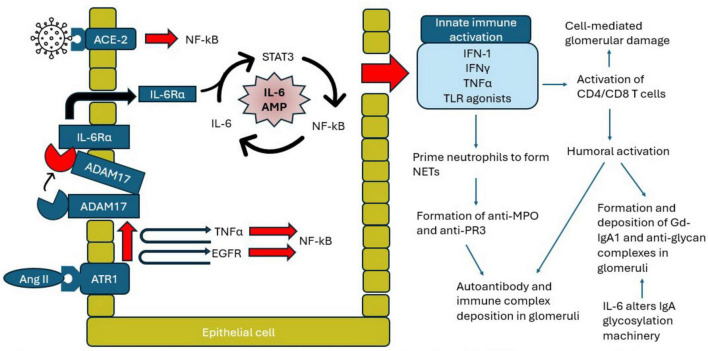
Proposed mechanism of the IL-6 amplifier pathway in the setting of coronavirus disease 2019 (COVID-19). Ang II, angiotensin II; ACE-2, angiotensin-converting enzyme 2; ATR1, angiotensin II type 1 receptor; ADAM17, disintegrin and metalloproteinase 17; TNF-a, tumor necrosis factor alpha; EGFR, epidermal-like growth factor receptor; NF-kB, nuclear factor kappa B; STAT3, signal transducer and activator of transcription 3; IL-6, interleukin 6; IL-6Ra, interleukin 6 receptor alpha; IFN-1, type 1 interferon; IFN-y, interferon gamma; TLR, toll-like receptor; NET, neutrophil extracellular traps; MPO, myeloperoxidase; PR3, proteinase 3; Gd-IgA1, galactose-deficient IgA1.

The reports of both c-ANCA (45, 189–194) and p-ANCA (16, 52, 53, 84, 101, 176, 195–208) GN following mRNA vaccination potentially suggests that SARS-CoV-2-associated NETs and AAV-GN are mediated by the virus’s S protein, or even the viral mRNA itself. Indeed, mRNA detection by dendritic cells and macrophages leads to increased activity of type 1 interferon (IFN-1) and other cytokines, which prime neutrophils to release reactive oxygen species and lytic enzymes, and facilitate the formation of NETs (Figure 3) (209).

**FIGURE 4:**
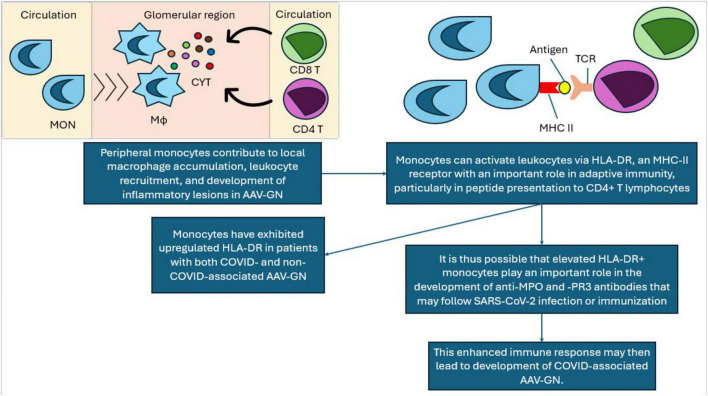
Proposed mechanism of MHC-II contribution to coronavirus disease (COVID)-associated AAV-GN. MON, monocyte; Mo, macrophage; CYT, pro-inflammatory cytokines; CD4 T, CD4+ T cell; CD8 T, CD8+ T cell; MHC II, major histocompatibility complex class II; TCR, T cell receptor; AAV-GN, ANCA- associated vasculitis and glomerulonephritis; HLA-DR, human leukocyte antigen receptor; MPO, myeloperoxidase; PR3, proteinase 3.

In addition to NET dysregulation, abnormal expression and activity of major histocompatibility complex (MHC) class II molecules is also implicated in AAV-GN pathogenesis, as outlined in Figure 4 (146, 184, 210–214).

#### 3.1.6 Lupus nephritis

Whether through clinical diagnosis or confirmed by biopsy, there have been a number of reports correlating SARS-CoV-2 infection with new-onset lupus nephritis (LN) (44, 215–222) as well as flares of chronic LN (96, 223–226) with or without systemic lupus erythematosus (SLE). Additionally, adenoviral vector and mRNA vaccines against SARS-CoV-2 have been associated with *de novo* (47, 52, 55, 78, 227, 228) and relapsed (48, 82, 229–232) LN and SLE. LN biopsies in COVID-19 are consistent with classic LN, which generally stains positive for deposits with IgG, C3, and C1q dominance (233). Such clinical and morphological findings contribute to the growing evidence of COVID-19 exacerbating underlying autoimmunity.

### 3.2 Cytokine-mediated glomerular damage

While an effective immune response to SARS-CoV-2 involves both B- and T cells, the rapid nature of GN onset post-COVID-19 vaccination implicates T cells as the more important mediators of glomerular damage (234). T cells respond to viral mRNA with rapid production of cytokines (e.g., IFN-gamma, tumor necrosis factor (TNF)-alpha, interleukin (IL)-6), which can both directly damage glomeruli as well as augment B cell activity and immune complex deposition (Figure 3) (234).

#### 3.2.1 Minimal change disease

Coronavirus disease 2019 has been correlated with podocytopathies that traditionally were thought to be driven by non-immune complex deposition processes. For example, COVID-19 infection has been associated with minimal change disease (MCD), a nephrotic syndrome previously thought to be cytokine-mediated (33, 35, 36, 43, 44, 96, 125, 235–237). Vaccination against the virus also may develop new-onset or relapsed MCD (16, 36, 47, 48, 52, 53, 55, 78, 82, 83, 237–258). While recent emerging evidence suggests that a number of MCD cases may in fact be driven by pathologic antibodies to nephrin (259), in the majority of cases, there remains a complex picture of interactions which likely reflect multiple parts of the immune system, including cytokine activity, T cell dysregulation, and B cell activation (260).

#### 3.2.2 Focal segmental glomerulosclerosis

Focal segmental glomerulosclerosis (FSGS) may similarly be driven in large part by cytokine damage rather than immune complex deposition. Additionally, recurrence or relapse of FSGS is thought to be potentially mediated by an unidentified circulating factor, whether it be an unidentified antibody, T cell cytokine, or non-immunogenic permeability factor, which could potentially be elicited by viruses such as SARS-CoV-2, leading to podocyte toxicity (Figure 2) (261, 262). FSGS has developed following the immune response elicited by SARS-CoV-2 infection (23, 44, 263–270) and vaccination (36, 52, 55, 78, 82, 271–274).

Collapsing FSGS (cFSGS), also known as collapsing glomerulopathy, is a morphological variant of FSGS. A plethora of cases have identified a temporal link between COVID-19 and cFSGS, particularly in the African-American population, with a higher incidence and poorer prognosis among high-risk apolipoprotein L1 (APOL1) variants (36, 37, 55, 125, 222, 235, 237, 271, 275–303).

Coronavirus disease-associated cFSGS has incited many comparisons to HIV-associated nephropathy (HIVAN). HIVAN appears to have a similar genetic predisposition, with higher incidence among African-Americans carrying APOL1 variants (304). More recently, non-HIV viruses, including hepatitis C virus (HCV) (305) and SARS-CoV-2 (304), have been implicated in its pathogenesis. Given their notable similarities, it may be fruitful to apply clinical knowledge of HIVAN pathogenesis and management toward COVID-associated cFSGS. Based on previous glucocorticoid use in HIVAN, one group of authors included prednisone in their treatment regimen for COVID-associated cFSGS, yielding successful outcomes (306). Such approaches may be useful moving forward, given that treatment for COVID-associated cFSGS is generally challenging with a poor prognosis (302).

It is important to note that whereas HIVAN is primarily driven by direct viral invasion and cytopathy of renal cells, this does not seem to be the case for COVID-associated FSGS (307). Many investigators propose that both collapsing and non-collapsing glomerulopathy in the setting of COVID-19 result from cytokine-mediated activation of innate and adaptive immune cells (263, 275, 282, 308). IFN pathways may be particularly important in COVID-associated cFSGS, as demonstrated by the presence of “IFN footprints” in some patients (96, 308). Previous research shows that IFN therapy may even induce cFSGS and other nephropathies (309, 310).

It is possible that there is a convergence in cytokine pathways between COVID-19 and other inflammatory states, such as in transplant immunity. Acute T cell-mediated allograft rejection has developed subsequent to SARS-CoV-2 infection and immunization, correlated with a CD3 + T cell predominance (96, 226, 237, 263). Furthermore, both IFN and granulocyte colony-stimulating factor–which are elevated in COVID-19 (311–313)–are important cytokines for T cell-mediated exacerbation of autoimmune GN (314) and acute transplant rejection (315).

Multiple allograft recipients have developed collapsing glomerulopathy in the setting of COVID-19 infection and immunization, with two patients expressing low-risk APOL1 alleles (48, 226, 263, 264, 279, 295, 316–321). Similarly, non-transplant patients have also presented with COVID-associated cFSGS without high-risk APOL1 polymorphisms; these patients had significant pre-existing conditions, including SLE and HIV (222, 291). It is possible that a pre-existing state of immune dysregulation (e.g., SLE, HIV, transplant immunity) may predispose or “prime” cytokine pathways to be triggered by COVID-19, resulting in cFSGS even in the absence of high-risk APOL1 mutations. Still, the presence of a high-risk APOL1 variant likely confers a worse prognosis, as exemplified by a high-risk APOL1 patient developing severe COVID-19 infection and cFSGS despite having prior immunity to SARS-CoV-2 (316).

Although the nephrotoxic effect of high-risk APOL1 variants is not fully understood, some proposed mechanisms include impaired endolysosomal trafficking, inflammasome activation, and APOL3 control of actomyosin in podocytes (322). The inflammatory response to COVID-19 may act as a “second hit” for APOL1 variants that at baseline are at high risk for developing glomerulopathy (275). Previously established second-hit triggers of glomerulopathies in high-risk APOL1 variants include HIV, SLE, and IFN therapy (323). SARS-CoV-2 is being considered as a second-hit trigger for cFSGS in a similar manner.

Innate immune pathways (e.g., IFN-1) can enhance APOL1 expression, and thus may facilitate COVID-associated cFSGS via upregulation of high-risk APOL1 alleles (Figure 2) (324–326). However, some cases of COVID-associated cFSGS in high-risk APOL1 variants did not exhibit IFN footprints (282, 285) or elevated IFN-1 expression (275). Additionally, Meliambro et al. did not find significant differences in APOL1 expression between controls and a high-risk APOL1 COVID-19 patient with c-FSGS (327). However, this may have been confounded by the anti-inflammatory action of hydroxychloroquine or the delayed kidney biopsy (327).

Overall, the literature demonstrates that COVID-associated cFSGS can develop in low, intermediate, or high-risk APOL1 individuals, with greater frequency and severity in the latter group and especially affecting those of African ancestry. Although IFN pathways are potentially implicated, knowledge gaps remain regarding the specific cytokines most strongly contributory to the glomerular injury of COVID-associated FSGS.

## 4 Hypothesized pathophysiology of COVID-associated glomerulonephritis

### 4.1 Controversies surrounding direct viral damage versus immune-mediated damage

Coronavirus disease-GN may arise via a variety of potential mechanisms. These include, but are not limited to: direct viral cytopathy, immune hyperactivation, hemodynamic instability, and metabolic imbalances (9). Researchers have attempted to tease out which underlying mechanisms contribute the most.

The first reported renal biopsy of a live COVID-19 patient showed collapsing glomerulopathy without signs of viral presence within renal cells (276). Many subsequent COVID-GN reports since then have similarly demonstrated a low likelihood that direct viral cytotoxicity is responsible for COVID-GN (27, 96, 275, 277, 278, 280, 328).

This may be the result of renal ACE-2 and TMPRSS2 expression. ACE-2 seems to be predominant in proximal tubules, whereas TMPRSS2 appears to be predominant in the distal tubules (329, 330). Given that ACE-2 and TMPRSS2 work in tandem with one another, their differential expression in separate regions of the nephron may limit SARS-CoV-2’s ability to enter.

Still, some authors maintain that SARS-CoV-2 infiltration into renal cells may cause COVID-GN via direct cytotoxicity and vasculitis (331, 332). Several cases observed particles within tubular epithelium and podocytes possibly suggestive of SARS-CoV-2; however, these particles may instead be clathrin-coated vesicles that are normally found within cells (22, 23, 235, 281, 318, 319, 333).

Some articles have also reported SARS-CoV-2 mRNA within kidney tissue (20, 96, 334). However, other biopsies have failed to demonstrate this (27, 110, 129, 277). Additionally, a number of COVID-GN cases occur post-infection, after viral clearance and negative PCR testing (17, 129, 138). COVID-GN has also developed following mRNA, adenoviral-vector, and inactivated vaccines, further demonstrating that direct viral infection is not required for nephropathy (47).

A final piece of evidence dissuading against direct virus-mediated COVID-GN is the presentation pattern. COVID-19 is associated with a wide spectrum of glomerular and tubular disease states, a pattern more consistent with systemic immune hyperactivation as opposed to direct viral cytotoxicity (96, 187). Overall, it appears unlikely that direct virus-mediated damage is predominant in COVID-GN.

### 4.2 Cytokine storm and IL-6 amplifier

Severe acute respiratory coronavirus infection is strongly associated with upregulated activity of CD4+ and CD8+ T cells, despite a decrease in the absolute number of CD8+ T cells (335, 336). COVID-19 vaccines can also stimulate robust CD4+/CD8+ responses with enhanced cytokine production (19, 337, 338). Furthermore, it has been established that such T cell dysregulation and hyperstimulation is linked to both immune complex-driven GN and cytokine-driven GN, including IgAN, LN, MCD, and FSGS (339–341).

We stress that a major pillar of COVID-GN is driven by cytokines, specifically a T cell-mediated cytokine storm. As summarized in Figure 3, current research implicates the IL-6 amplifier system in propagating the cytokine storm of COVID-19 (13, 337, 342–346). The age-dependent strength of the IL-6 amplifier’s feedback loop may correspond with the age-dependent increase in COVID-19 morbidity and mortality (347).

Interleukin-6-mediated lymphocyte recruitment may play a role in the humoral and cell-mediated processes underlying COVID-GN. Studies have found elevated IL-6 levels in the serum of patients with COVID-related IgAN and vasculitis (25, 27). Furthermore, IL-6 may strongly alter IgA glycosylation machinery and significantly contribute to glomerular IgA deposition (348–350). It is possible that the IL-6 amplifier provoked by SARS-CoV-2 promotes the production of Gd-IgA1, resulting in complex deposition within the glomeruli. IFN-1 cytokines, specifically IFNa, may further contribute to COVID-associated flares of chronic IgAN (351, 352). Increased phosphorylation and activation of STAT3 has also been observed in COVID-GN (327). Targeting the expression or activation of factors like STAT3, IFN-1, and IL-6 may hold therapeutic potential for COVID-GN.

It is likely that the IL-6 feedback loop and cytokine storm in COVID-19 are enhanced by complement pathways. Complement activation has shown to contribute to SARS-CoV-2 pathogenesis and severity (353). COVID-19 patients have elevated serum levels of complement proteins like C5a, which can boost IL-6 and TNFa expression (353–355). Similarly, COVID-GN can also present with elevated C5a levels, increased C5aR activity, and decreased C3/C4 levels (356). It is thus possible that complement pathways propagate COVID-GN via potentiation of cytokine activity.

Overall, although the exact relationship remains unclear, there is mounting evidence that COVID-GN involves an IL-6 amplifier-driven hyperinflammatory storm.

### 4.3 SARS-CoV-2 superantigen

Cheng et al. (357) found that SARS-CoV-2’s S protein has superantigen characteristics, exhibiting high affinity for complementarity determining regions of both α- and β-chain variable domains of T cell receptors (TCRs), and that this interaction is further strengthened by certain mutations in the virus’s genome.

Researchers have also found that there is a skew in expression of TCR repertoire among COVID-19 patients with hyperinflammation (358). These effects seem to be due to a sequence motif within S’s binding epitope that is unique to SARS-CoV-2; this motif is absent in other coronaviruses and instead resembles bacterial superantigens in both sequence and structure (358). Enterotoxin B and C of staphylococci can act as superantigens, inducing GN via mass stimulation of T cell cytokines and polyclonal immunoglobulin (359, 360). SARS-CoV-2’S protein may act as a superantigen in a similar manner, driving the development of GN via a cytokine storm.

### 4.3 Interactions with the renin-angiotensin-aldosterone system

Interactions with RAAS may contribute to the cytokine storm in COVID-19. Binding of SARS-CoV-2 to ACE-2 results in increased angiotensin II (Ang II) binding to angiotensin II type 1 receptor (ATR1), resulting in the induction of disintegrin and metalloproteinase 17 (ADAM17), which not only generates the mature forms of epidermal growth factor receptor (EGFR) and TNF-a, but also processes the membrane form of IL-6 receptor alpha (IL-6Ra) into its soluble form (3, 361–363). The net downstream effect is IL-6 amplifier activation (Figure 3). COVID-GN thus may involve dysregulation of the ACE-2 and Ang II axis.

However, conflicting findings have been reported, summarized in Table 1 (346, 364–375). Interestingly, in one COVID-19 patient with cFSGS, there was no significant difference in ACE-2 transcript expression compared to normal kidney samples (327). However, translational and post-translational modifications of ACE-2 can cause discrepancies between transcript and protein expression (371, 376).

**TABLE 1:** Opposing views with seemingly conflicting evidence in the literature regarding the role of angiotensin-converting enzyme-2 (ACE-2) expression and renin-angiotensin-aldosterone system (RAAS) in the development and severity of coronavirus disease-associated glomerulonephritis (COVID-GN).

Hypothesis and rationale: decreased ACE-2 expression correlates with worse outcomes in COVID-19 and COVID-GN, due to greater Ang II activity and inflammation	Hypothesis and rationale: increased ACE-2 expression correlates with worse outcomes in COVID-19 and COVID-GN, due to greater receptor availability for SARS-CoV-2 to enter target cells
In normal state, ACE-2 is counter-regulatory to RAAS. ACE-2 opposes ACE-mediated vasoconstriction and inflammation, particularly in the kidneys, which have an even higher expression of ACE-2 than the lungs.	Binding of Ang II to ATR1 can markedly increase ACE-2 expression, which resulted in enhanced SARS-CoV-2 cell entry. This was reversed by irbesartan, an ATR1 antagonist.
An increased ACE/ACE-2 ratio has been correlated with greater disease severity in COVID-19 patients.	There was elevated ACE-2 expression in the lungs of patients with comorbidities that confer high risk for severe COVID-19.
ACE-2 knockout in animals worsens both hypertensive nephropathy and inflammatory lung lesions. These lesions were attenuated by recombinant ACE-2 and angiotensin II receptor blocker (ARB).	–

There is a shortage of conclusive literature, but the current evidence seems to suggest that decreased ACE-2 expression predisposes patients to severe COVID-19 and COVID-GN. Regardless, ACE-2 receptors should be further investigated as a potentially crucial component of COVID-GN pathogenesis.

## 5 Discussion and conclusion

The overall literature seems to suggest that there may be an association between COVID-19 and both *de novo* and relapsed GN. The most common overall type of COVID-GN was FSGS, particularly cFSGS. In infection, the most common cases were *de novo* FSGS followed by *de novo* AAV-GN. In vaccination, the most common cases were relapsed IgAN followed by *de novo* AAV-GN. IgAN had the most relapse cases in both infection and vaccination, potentially suggesting a greater prominence of “unmasking” mechanisms for COVID-associated IgAN.

Prognosis seems to differ between different GN pathologies–patients who developed COVID-associated IgAN or MCD were generally more likely to recover kidney function with appropriate management (55).

The numerous instances of mixed glomerular histologies (e.g., MN and cFSGS, IgAN, and AAV-GN) potentially suggests a multifactorial pathophysiology for COVID-GN (37, 49, 55, 60). Cytokine storms, which may or may not be accompanied by immune complexes, is the most consistently prevalent mechanism described within the literature. SARS-CoV-2 infection and immunization likely induce robust CD4+ upregulation, ensuing the IL-6 amplifier, which is enhanced by RAAS and complement pathways. Cytokines of interest include IL-6, IFN-1, and TNFa, which may mediate polyclonal immunoglobulin stimulation and/or direct cytotoxicity of glomeruli. Targeting these pathways may prove fruitful in our understanding of COVID-19 disease and treatment.

Genetic interactions with SARS-CoV-2’s inflammatory cascade can predispose certain individuals to developing severe COVID-19 and GN. Mutations and polymorphisms in ATR1, ACE-2, and APOL1 should be further investigated as potential predisposing factors for COVID patients.

There was a higher number of GN cases associated with COVID-19 vaccines than infections in the literature (Figure 1). However, it is unlikely that vaccination confers a greater risk of GN. Vaccine cases are generally more controlled environments than active COVID-19, making them easier to study and report. Furthermore, multiple large population-wide studies across different countries have found that the COVID-19 vaccine rollout did not significantly increase overall incidence of glomerular disease (377, 378). Among the different types of COVID-19 vaccines, mRNA vaccines were the most commonly reported in association to GN. This does not necessarily indicate that mRNA vaccines inherently carry a higher propensity for GN, but rather, likely reflects the widespread availability of mRNA vaccines for COVID-19 across the global population.

Although this review has aimed to be fully comprehensive, there are limitations. Due to the retrospective nature intrinsic to systematic reviews, we are unable to definitively claim a causative effect between COVID-19 and GN. There is a possibility that some COVID-GN cases were due to concurrent disease or incidental timing. For example, one case of AAV, not included in this review, was initially believed to be secondary to COVID-19 vaccination, until underlying Dengue infection was identified (379, 380). This review also includes both case reports and national or international registries, resulting in a slight theoretical possibility of “double-counted” cases.

Given the novel nature and ever-developing literature surrounding SARS-CoV-2 pathophysiology, it is not surprising that COVID-GN presents with unsettled results. For example, precise etiology is difficult to ascertain in dual diagnosis cases (e.g., AAV-GN and anti-GBM) (99). It can also be difficult to distinguish new-onset AAV in COVID-19 patients, due to pulmonary and vasculitis-like symptomatology sometimes resembling one another (381–384).

It was unclear if certain COVID-GN cases were acute or chronic processes. Some patients had a prior history of microscopic hematuria, yet due to their subclinical nature, they were never biopsied to confirm a GN diagnosis (60, 80). Upon exposure to SARS-CoV-2 infection or immunization, they developed new-onset gross hematuria and/or AKI, prompting a renal biopsy, leading to a GN diagnosis (60). This review tallied such cases as relapses–although the GN diagnosis was new, the patient history suggested an “unmasking” of underlying pathology.

This review specifies particular T cell types, antibodies, cytokines, and genes as requiring further investigation. However, other avenues (e.g., regulatory T cell inhibition) are also underexplored mechanisms for COVID-GN. The pathogenesis of COVID-GN is most likely multifactorial, involving multiple pathways of the immune system, possibly with secondary systemic effects (e.g., hypoxia).

This review is restricted by the retrospective evaluation of the limited number of cases published between 2020 and 2023. Continued attention toward COVID-GN may elucidate greater facets of its pathophysiology and treatments.

## Data Availability

The original contributions presented in this study are included in this article/supplementary material, further inquiries can be directed to the corresponding author.
